# Increase in invasive group A streptococcal infections (iGAS) in children and older adults, Norway, 2022 to 2024

**DOI:** 10.2807/1560-7917.ES.2024.29.20.2400242

**Published:** 2024-05-16

**Authors:** Beatriz Valcarcel Salamanca, Pascale Renée Cyr, Yngvild Emblem Bentdal, Sara Viksmoen Watle, Astrid Louise Wester, Åse Marie Wikman Strand, Håkon Bøås

**Affiliations:** 1 Department of Infection Control and Vaccines, Norwegian Institute of Public Health, Oslo, Norway; 2 ECDC Fellowship Programme, Field Epidemiology path (EPIET), European Centre for Disease Prevention and Control (ECDC), Stockholm, Sweden; 3 Department of Infectious Disease Registries, Norwegian Institute of Public Health, Oslo, Norway; 4 Department of Bacteriology, Norwegian Institute of Public Health, Oslo, Norway

**Keywords:** Group A Streptococcus, iGAS, invasive, outbreak, Streptococcus pyogenes

## Abstract

At the end of 2022 and most notably during the first half of 2023, the number of invasive group A streptococcus (iGAS) notifications increased in Norway, largely affecting children younger than 10 years, as observed in several other countries. Following this atypical season, a new surge in the number of iGAS notifications began in December 2023 and peaked between January and February 2024, now particularly affecting both children younger than 10 years and older adults (70 years and above).

During the first half of 2023, an increase in invasive group A streptococcus (iGAS) notifications was observed in Norway, followed by a new surge in early 2024. Although most frequently associated with mild illness such as sore throat and tonsillitis (strep throat), *Streptococcus pyogenes* (group A *Streptococcus* or GAS) infections can also cause severe invasive and life-threatening outcomes such as sepsis or necrotising fasciitis. In Norway, bloodstream GAS infections have been mandatorily notifiable to the Norwegian Surveillance System for Communicable Diseases (MSIS) since 1977 [1], whereas all invasive GAS (iGAS) infections have been notifiable since 1993 [2] and all GAS detected in sterile material samples since 1995 [3].

In order to better assess changes in incidence, age distribution and clinical presentation of cases, we aimed to evaluate the epidemiological characteristics of iGAS cases reported in Norway during the last 2 years and compare them with cases reported before the COVID-19 pandemic.

## Notification of invasive group A streptococcal infections

**Figure 1:**
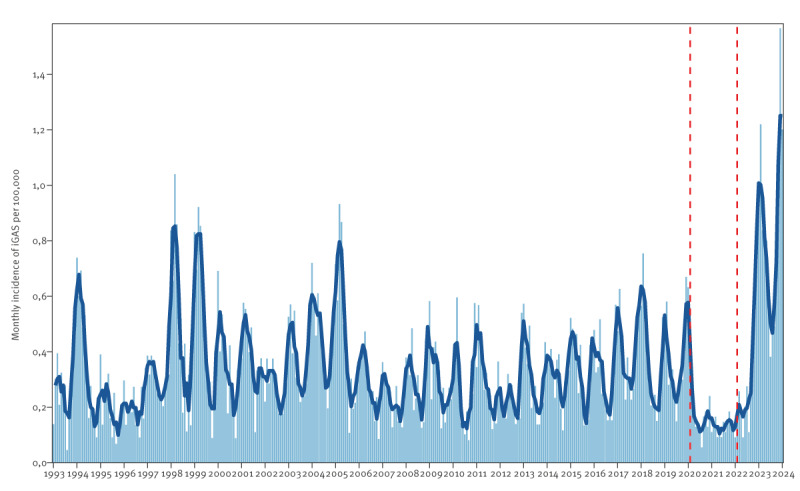
Monthly incidence of invasive group A streptococcal infection for all ages, Norway, January 1993– February 2024 (n = 6,219)

The historical overall monthly incidence of iGAS in Norway from January 1993 to February 2024 is shown in Figure 1. The incidence in Norway has been remarkably high since the end of 2022 compared with the years before the COVID-19 pandemic, and it peaked in January 2024. We applied for data access by the end of February 2024. Data extraction for all data sources presented here occurred in early March. To account for reporting delay, we limited the data presentation to end of February 2024. To allow comparison between similar time periods and to account for the COVID-19 pandemic control measures, we defined four time periods: pre-pandemic period (March 2015 to February 2020), COVID-19 pandemic period (March 2020 to February 2022), late-pandemic period (March 2022 to February 2023) and post-pandemic period (March 2023 to February 2024). We estimated the expected and excess incidence for the late-pandemic and post-pandemic periods, using generalised linear regression and 10 pre-pandemic years (2010 to 2019) as baseline. We append details on the methodology used for estimation of the expected and excess incidence in the Supplement.

Light blue bars indicate monthly incidence. The dark blue line represents three-month moving averages. Vertical red dotted lines represent the start and end of the COVID-19 pandemic.

In total, 2,129 cases were reported to MSIS between March 2015 and February 2024. On average, 230 cases were reported annually from March to February during the pre-pandemic period (standard deviation (SD): 22), corresponding to 4.3 cases per 100,000 population (SD: 0.39) (Table 1). During the COVID-19 pandemic, there were 101 cases on average (SD: 14), corresponding to 1.9 per 100,000 population (SD: 0.27). During the late-pandemic period, 243 cases were reported (4.5 cases per 100,000 population), followed by a large increase to 553 cases (10 cases per 100,000 population) between March 2023 and February 2024 with an estimated excess of 266 cases (95% prediction interval (PI): 212–320), corresponding to 4.8 excess cases per 100,000 population (95% PI: 3.8 – 5.8). Supplementary Figure S1 contains additional details on the estimated expected and excess annual incidence.

**Table 1:** Key demographic characteristics of all invasive group A streptococcus notifications to MSIS, Norway, March 2015–February 2024 (n = 2,129)

Characteristics	Pre-pandemic^a ^(n = 1,136)		Pandemic^b ^(n = 197)		Late/post-pandemic^c^			
					2022/2023 (n = 243)		2023/2024 (n = 553)	
Annual number of cases (SD)	230^d^ (22)		101^d^ (14)		243		553	
Annual incidence/100,000 (SD)	4.3^d^ (0.39)		1.9^d^ (0.27)		4.5		10	
Median age in years (IQR)	61 (37–74)		51 (34–73)		51 (29–72)		58 (33–73)	
	n	%	n	%	n	%	n	%
Sex								
Female	557	49	90	46	101	42	250	45
Male	579	51	107	54	142	58	303	55
Age group in years								
0–9	105	9.2	13	6.6	49	20	85	15
10–19	28	2.5	6	3.0	5	2.1	21	3.8
20–49	291	26	75	38	65	27	116	21
50–69	316	28	45	23	56	23	137	25
≥ 70	396	35	58	29	68	28	194	35
Region								
North	99	8.7	13	6.6	10	4.1	34	6.1
Central	118	10	19	9.7	23	9.5	42	7.6
West	258	23	28	14	58	24	143	26
South	53	4.7	11	5.6	10	4.1	32	5.8
East	609	54	125	63	142	58	298	54

iGAS: invasive group A streptococcus; IQR; interquartile range; MSIS: the Norwegian Surveillance System for Communicable Diseases; SD: standard deviation.

^a ^Pre-pandemic period from 1 March 2015 to 28 February 2020.

^b^ Pandemic: COVID-19 pandemic period from 1 March 2020 to 28 February 2022.

^c ^Late/post-pandemic period from 1 March 2022 to 29 February 2024.

^d^ Annual average.

**Figure 2:**
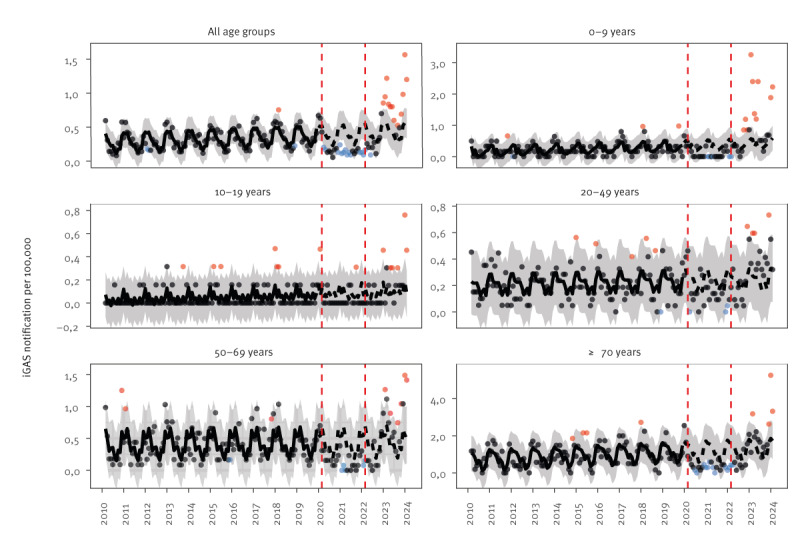
Expected and observed monthly invasive group A streptococcus notification rates per 100,000 population by age group and study periods, Norway, March 2010–February 2024 (n = 3,011)

At closer examination of the monthly notifications in each period, we observed a rise starting in December 2022. This surge was most noticeable throughout 2023 and early 2024. We estimated that from January 2023 to February 2024, all months except August and October had a statistically significant excess of notifications. The largest excess was observed in January 2024 with an almost threefold increase in the number of cases (Figure 2).

iGAS: invasive group A streptococcus; PI: prediction interval.

Black dots indicate monthly iGAS notification rates during the baseline period 2010–2019. The vertical red dotted lines represent the start and end of the COVID-19 pandemic period. Shaded band represents the estimated expected notification rates range (95% prediction interval). Blue dots represent annual rates below the lower limit of 95% PI. Red dots represent annual rates exciding the upper limit of 95% PI.

We did not find any statistically significant differences in the geographical distribution of cases when comparing the periods before and after the pandemic.

## Sex and age distribution of iGAS notifications

There was a significantly higher proportion of cases in males during the late/post-pandemic periods (445/796; 56%) compared with the pre-pandemic period (579/1,136; 51%; p = 0.03). This observation can be attributed to the uneven age distribution of cases in males, characterised by higher male-to-female case ratios in children aged 0–9 years in the late-pandemic period (69%) and in adults over 50 years in the post-pandemic period (ca 60%). In Supplementary Figure S2, we provide additional detail on the proportion of cases by sex and age group for different time periods.

During the late-pandemic and post-pandemic period, respectively 20% and 15% of cases were in 0–9-year-old children. This represents a substantial increase when compared with the pre-pandemic period where the average was 9.2% (p < 0.001). The main surge among 0–9-year-olds occurred between February to April 2023, with the largest peak in February 2023 (Figure 2). We observed a smaller but considerable increase in January and February 2024. We estimated excess cases among these children to be 23 (95% PI: 16–31) between March 2022 and February 2023 and 58 (95% PI: 50–66) between March 2023 and February 2024. The increase in paediatric cases was seen throughout Norway.

In January 2024, a pronounced peak of 38 cases occurred in people ≥ 70 years, corresponding to a threefold increase in monthly incidence when compared with the same month in previous periods, and corresponding to an excess of 74 cases (95% PI: 44–105) between March 2023 and February 2024.

## Clinical presentation

Data on clinical presentation and suspected portal of entry were obtained from the MSIS register. For 792 (99.5%) of the cases notified during either of the late/post-pandemic periods, data were checked against information found in the MSIS Laboratory database for quality control purposes. For details on the quality control exercise performed we refer to section *Data on clinical presentation and portal of entry of infection* in the Supplement. Of 658 cases older than 10 years reported in the late/post-pandemic periods, skin/sores and respiratory tract were the suspected portal of entry for 29% and 20% of cases, respectively. Of these cases, 313 (48%), were clinically severe (Table 2) and 36 (5.5%) were registered with a fatal outcome. Bacteraemia (n = 544; 83%), sepsis (n = 267; 41%), skin-related infections (n = 92; 14%) and necrotising fasciitis (n = 76; 12%) were the most reported clinical manifestations. Of all cases, 231 (35%) presented bacteraemia with confirmed clinical sepsis and 37 (5.6%) presented bacteraemia with organ failure/septic shock. Nine cases (1.4%) were reported with skin infection and progressed to necrotising tissue. No significant difference was observed between the two periods (Table 2).

**Table 2:** Clinical presentation and suspected portal of entry of invasive group A streptococcus for cases 0–9 years and ≥ 10 years, Norway, March 2022–February 2024 (n = 792)

Characteristic		2022/2023			2023/2024			Overall		
		n	%		n		%	n	%	
Age ≥ 10 years		n = 192			n = 466			n = 658		
Portal of entry of infection	Obstetric/gynaecologic	7		3.6	23	4.9		30		4.6
	Respiratory infection	43		22	90	19		133		20
	Skin/wound infection	50		26	139	30		189		29
	Unknown	86		45	198	42		284		43
	Other^a^	6		3.1	16	3.4		22		3.3
Clinically severe cases^b^		89		46	224	48		313		48
Clinical manifestations	Bacteraemia	162		84	382	82		544		83
	Sepsis	73		38	194	42		267		41
	Skin infection	22		11	70	15		92		14
	Necrotising fasciitis	20		10	56	12		76		12
	Organ failure/septic shock	13		6.8	35	7.5		48		7,3
	Pleuritis and/or empyema	8		4.2	13	2.8		21		3.2
	Bone infection	16		8.3	44	9.4		60		9.1
	Meningitis	1		0.5	5	1.1		6		0.9
Clinical manifestations, combinations	Bacteraemia with sepsis	65		34	166	36		231		35
	Bacteraemia with sepsis and organ failure/septic shock	10		5.2	27	5.8		37		5.6
	Skin infection with necrotising fasciitis	1		0.5	8	1.7		9		1.4
Age < 10 years		n = 49			n = 85			n = 134		
Portal of entry of infection	Respiratory infection	26		53	41	48		67		50
	Skin/wound infection	8		16	12	14		20		15
	Unknown	12		24	25	29		37		28
	Other^a^	3		6.1	7	8.2		10		7.5
Clinically severe cases^a^		20		41	50	59		70		52
Clinical manifestations	Bacteraemia	24		49	54	64		78		58
	Sepsis	13		27	29	34		42		31
	Skin infection	3		6.1	5	5.9		8		6.0
	Necrotising fasciitis	1		2.0	10	12		11		8.2
	Organ failure/septic shock	2		4.1	7	8.2		9		6.7
	Pleuritis and/or empyema	10		20	23	27		33		25
	Bone infection	6		12	10	12		16		12
	Meningitis	2		4.1	1	1.2		3		2.2
Clinical manifestations, combinations	Bacteraemia with sepsis	7		14	22	26		29		22
	Bacteraemia with sepsis and organ failure/septic shock	1		2.0	7	8.2		8		6.0
	Skin infection with necrotising fasciitis	0		0	2	2.4		2		1.5

^a ^Other: urinary tract, ear, eye and dental infections, mother-to-child transmission and through medical equipment.

^b ^Clinically severe: cases reported as having at least one of the following clinical presentations: sepsis, necrotising fasciitis, organ failure/septic shock, pleuritis and/or empyema, meningitis.

Of cases in children 0–9 years (n = 134), 70 (52%) were clinically severe with a significantly higher proportion of severe cases in the second period (50/85; 59%) compared with the first (20/49; 41%) (p = 0,04). Contributing to this was the large increase of cases with necrotising fasciitis (from 2% (1/49) to 12% (10/85)), pleuritis and/or empyema (from 20% (10/49) to 27% (23/85)) and organ failure/septic shock (from 4.1% (2/49) to 8.2% (7/85)). In addition to bacteraemia and sepsis, pleuritis and/or empyema was the most reported clinical manifestation in children 0–9 years (33/134; 25%). Respiratory tract was the suspected portal of entry for 50% (n = 65) of cases 0–9 years.

## emm type distribution

Molecular typing data (*emm *type) have been available since 2019 and for 649 (30%) of the registered cases; for the total number of isolates with *emm* type data in each time period, we refer to the appended Supplementary Table S2. A total of 49 unique *emm* types were detected. In the pre-pandemic year (March 2019–February 2020), the most dominant *emm *type was *emm-*1 (46/129; 36%), followed by *emm-*28 (17/129; 13%). During the pandemic years, *emm-*89 was most dominant (29/167; 17%), followed by *emm-*1 (24/167; 14%). During the late/post-pandemic periods, *emm-*1 (116/353; 33%) and *emm-*12 (86/353; 24%) were most dominant; the detailed proportion of *emm* type data are appended in Supplementary Figure S3. Among clinically severe cases, *emm*-typing data were available for 179/383 (47%). No statistical association was found between the *emm*-types and clinical presentation; the detailed distribution of *emm* type by clinical presentation is appended in Supplementary Figure S4.

## Primary care consultations for strep throat infection

Primary care consultations for strep throat (international classification of primary care (ICPC) code R72) followed a similar pattern as iGAS notifications; graphs presenting the monthly iGAS notifications and monthly primary care consultations for strep throat can be found in Supplementary Figure S5. From the last quarter of 2022 and throughout 2023, the overall number of consultations for strep throat was approximately twofold higher than during the pre-pandemic years 2015 to 2019. This increase was most notable among children 0–14 years and adults 30–64 years, with a peak of consultations in December 2023 for children 0–4 years at approximately twice the level of the period 2015 to 2019. In the first quarter of 2024, the overall number of consultations for strep throat infections was lower compared with the same period in 2023, but still elevated compared with the pre-pandemic years.

## Discussion

In Norway, a strong increase in iGAS cases started in December 2022, continued throughout 2023 and reached the highest peak since year 1977 in January 2024. This increase occurred in all age groups but most notably in children (0–9 years) and older adults (≥ 70 years). The largest rise in children 0–9 years occurred in early 2023 and in early 2024 in older adults (≥ 70). We also observed an increase in disease severity among 0–9-year-olds. In line with our results, a larger proportion of male iGAS cases had previously been reported in Norway [2] and other countries [4,5], suggesting a potentially higher risk for males. Primary care consultations for strep throat also increased during the late/post-pandemic years, following a similar pattern as iGAS notifications.

Since December 2022, several European [4,6,7] and non-European [5,8] countries have documented an increase in iGAS infections, particularly in the paediatric population but also in older age groups [9-11]. The reason for this global rise is still unclear. A shift between *emm* types could have explained, in part, the increase in numbers and severity; however, no novel *emm* type has been observed in Norway. However, the sequencing of isolates was reduced in the post-pandemic period and is an important limitation to our findings.

Infection control measures implemented in Norway clearly impacted the exposure to GAS, as reflected in the low number of strep throat consultations and iGAS notifications during the pandemic years. This reduced exposure affected all age groups but especially children (0–9 years) and older population (≥ 70 years), with a lower number of iGAS cases than expected during the pandemic periods. The existence of a larger proportion of susceptible people due to reduced exposure could explain the large increase of paediatric cases in early 2023. In addition, the rebound of predisposing viral infections such as influenza, respiratory syncytial virus and varicella zoster virus after 2 years of reduced circulation, combined with frequent COVID-19 reinfections [12,13], may have increased individuals’ susceptibility to GAS infection and affected the immunologically naïve (0–4 years) and compromised (≥ 70 years) population the most.

## Conclusion

The ongoing increase in iGAS notifications globally, particularly in children and older adults, is of concern. To understand the main factors driving these changes and to effectively identify which public health interventions would aid in halting the rise requires further research. Continued monitoring of the epidemiological situation at the national and international levels is essential to detect new surges and identify potential changes in *emm* types and other bacterial characteristics associated with severe presentation of GAS infections. Clinicians should be on alert and familiar with recommendations for a prompt identification and treatment of GAS to minimise the risk of complications and reduce further transmission.

## Supplementary Data

990000 Supplement
