# Supplementary material for: Increase in invasive group A streptococcal infections (iGAS) in children and older adults, Norway, 2022 to 2024
Source: Euro Surveill. 2024 May 16;29(20):2400242. doi: 10.2807/1560-7917.ES.2024.29.20.2400242 (PMC11100296; doi:10.2807/1560-7917.ES.2024.29.20.2400242)
Supplement: Supplement [file 24-00242_SALAMANCA_Supplement.pdf]

Supplementary Material

"This supplementary material is hosted by *Eurosurveillance* as supporting information alongside the article [Increase in invasive group A streptococcal infections (iGAS) in children and older adults in Norway 2022-24], on behalf of the authors, who remain responsible for the accuracy and appropriateness of the content. The same standards for ethics, copyright, attributions and permissions as for the article apply. Supplements are not edited by *Eurosurveillance* and the journal is not responsible for the maintenance of any links or email addresses provided therein."

Data on clinical presentation and portal of portal of entry of infection

Data was obtained from the MSIS register and for cases notified during the periods 2022/23-2023/2024 (792/2140 cases), clinical records were checked against information found in the MSIS-Labdatabase for quality assurance purposes (according to MSIS-regulation § 2-6). Tests positive for GAS taken within a 3-week period after the date of notification to MSIS were considered suitable in the quality assurance exercise. Supplementary information about the portal of entry of infection and clinical presentation could be obtained from the MSIS-Labdatabase. Information was retrieved from test results or clinical information provided to the laboratories as indication for testing. Information was extracted if no clinician had reported relevant information to MSIS as mandated (according to MSIS-regulation § 2-1).

Mandatory clinical reports were not obtained for 310/792 cases (39%) that were reported to MSIS in the period between March 1*st*, 2022 to March 1*st*, 2024. In addition, clinical reports received did not always hold information about the clinical presentations that are codified in the MSIS variable, leaving 392 (49.5%) cases with no information (coded as unknown/other). After linking the data to the MSIS-Labdatabase and perusing for errors in clinical reports (relevant information provided in the wrong fields), all cases were assigned clinical information, but 147 (19%) had no information beyond a positive blood sample (bacteremia) or biopsy. The updated variable included a wider set of clinical presentations and combinations than was previously codified. It was then possible to assign cases into each

relevant subsets of clinical presentations they belonged to, with the possibility of belonging to one or more of the following groups: sepsis, necrotizing fasciitis, septic shock (including toxic shock syndrome) and organ failure, bacteremia (with clinical sepsis, with other severe symptoms than sepsis, with only mild symptoms, or with unknown clinical presentation), skin infections excluding necrotizing fasciitis (erysipelas, cellulitis and deep/soft tissue infections), pleuritis or empyema, bone infections (including arthritis, bursitis and osteomyelitis) and finally meningitis or encephalitis. Other possible clinical presentations not included in Table 2 were: abscess, cystitis, endocarditis, endometritis (or pelvic infection/inflammatory syndrome), epiglottitis, ethmoiditis (acute), eye infection, lymphadenitis, mastoiditisand/or inner ear infection, nephritis, orchitis, peritonitis, pneumonia, prosthetic infection, synovitis, tonsilitis, vaginitis, and finds in tissues post-mortem.

The MSIS-registry does not have a variable that encodes the point of entry of infections, nor does it require clinicians to specifically report this information. However, many clinicians report this information in open text field when requesting laboratory testing or report it indirectly through an analogous variable, route of transmission, which was available for 137 (17%) cases. After linking the data to the MSIS-Labdatabase and perusing clinical reports, information about the suspected point of entry was generated for 461 (58%) of cases.

Statistical analyses

To estimate the expected yearly iGAS incidence for the late-pandemic seasons 2022/23 and 2023/24, we fit a linear regression model with rates per 100 000 population as the outcome variable and year/season as covariate using data from ten pre-pandemic seasons (2010/11 to 2019/20). To estimate the expected monthly incidence, calendar months were also included in the model as covariates. Each age group were modelled independently. We estimated the 95% prediction interval for seasons 2022/23 to determine the threshold for excess incidence. The excess in iGAS cases was defined as the difference between the observed incidence and estimated upper bound of 95% prediction interval. All analyses were conducted using the R statistical software from the R Foundation.

Emm-typing

All isolated are identified and characterized by whole genom sequencing (WGS) at the National reference laboratory at the Norwegian Institute of Public Health. The DNA is first extracted from a bacterial culture using QiaCube (Qiagen) before the DNA is prepared using xGen DNA Library preparation EZ kit (integrated DNA Technologies) and then sequenced using Illumina sequencing platforms. WGS mappes nucleobases in the entire genome. Allelic definition for the characterization of emm-type (M-protein) is retrieved from CDC-database.

Discussion regarding emm-types

The most commonly identified emm-types in Norway were emm 1 and emm 12, with an increase in the proportion of emm 12 during the late/post-pandemic periods. This reflects what has been reported from other European countries [1]. Even though we see an increase in emm 12 in Norway, both emm types circulated before the pandemic. In May 2023 ECDC reported that the increase was not attributable to specific emm types or increased antibiotic resistance. However, the available data is insufficient to explore further if there has been a shift towards more toxicogenic variants like the $M1_{UK}$ or the $M1_{DK}$ lineage, as has been seen in other countries [2,3].

1. Communicable-Disease-Threats-Report-2-8-April-2023-Week-14 (europa.eu)

2. Eurosurveillance | Increase in invasive group A streptococcal infections and emergence of novel, rapidly expanding sub-lineage of the virulent Streptococcus pyogenes M1 clone, Denmark, 2023

3. Group A Streptococcal Meningitis with the M1UK Variant in the Netherlands - PMC (nih.gov)

Table S1. Key demographic characteristics of invasive group A streptococcus (iGAS) notifications of children 0-9 years of age, Norway, 1 March 2015 – 29 February 2024.

					Late/Post-pandemic[3]			
**Characteristics**	**Pre-pandemic[1]**		**Pandemic[2]**		**2022/2023**		**2023/2024**	
**Annual number of cases (mean*, SD**)**	22*	4**	7*	2**	49		85	
**Annual incidence per 100,000 (mean*, SD**)**	3.5*	0.6**	1,4*	0.4**	8		14	
**Age in years (median; IQR)**	4	2 - 6	4	2 - 5	2	1 - 5	3	1 - 6
**Sex (N, %)**								
Female	48	46	4	31	15	31	37	44
Male	57	54	9	69	34	69	48	56
**Region (N, %)**								
North	9	8.6	0	0	2	4.1	8	9,4
Central	7	6.7	3	23	3	6.1	7	8,2
West	33	31	5	38	14	29	22	26
South	4	3.8	1	7.7	1	2	4	4.7
East	52	50	4	31	29	59	44	52

[1]Pre-pandemic period from 1st March 2015 to 28th February 2020. [2] Pandemic: COVID19 pandemic period from 1st March 2020 to 28th February 2022. [1]Late/Post-pandemic period from 1st March 2022 to 29th February 2024. SD: standard deviation. IQR; interquartile range. N; total number of cases. %; proportion of the total number of case.

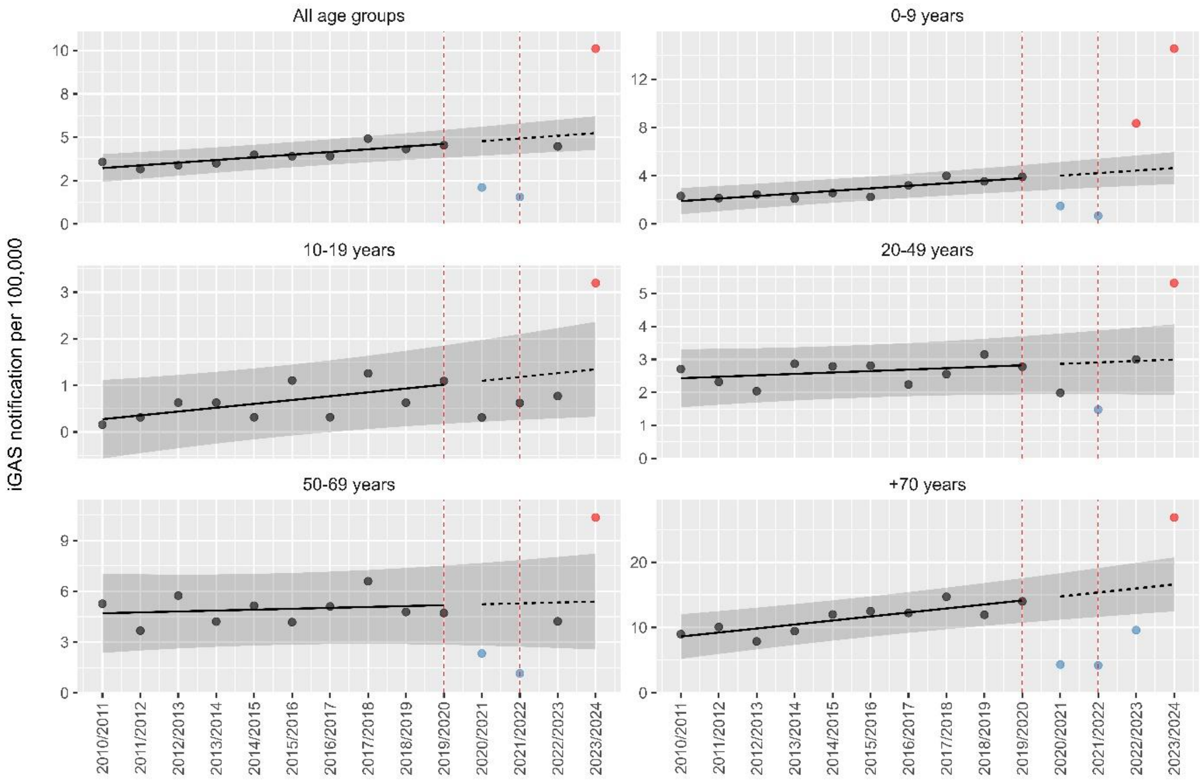

Figure S1. Expected and observed annual invasive group A streptococcus (iGAS) notifications notification rates per 100,000 population by age group and study periods. Baseline period 2010/2011-2019/2020. Black dots indicate observed annual notification rates during baseline period. Vertical red dotted lines represent the start and end of the pandemic period. Shaded band represents the estimated expected notification rates range (95% prediction interval). Blue dots represent annual rates below the lower limit of 95% PI. Red dots represent annual rates exceeding the upper limit of 95% PI.

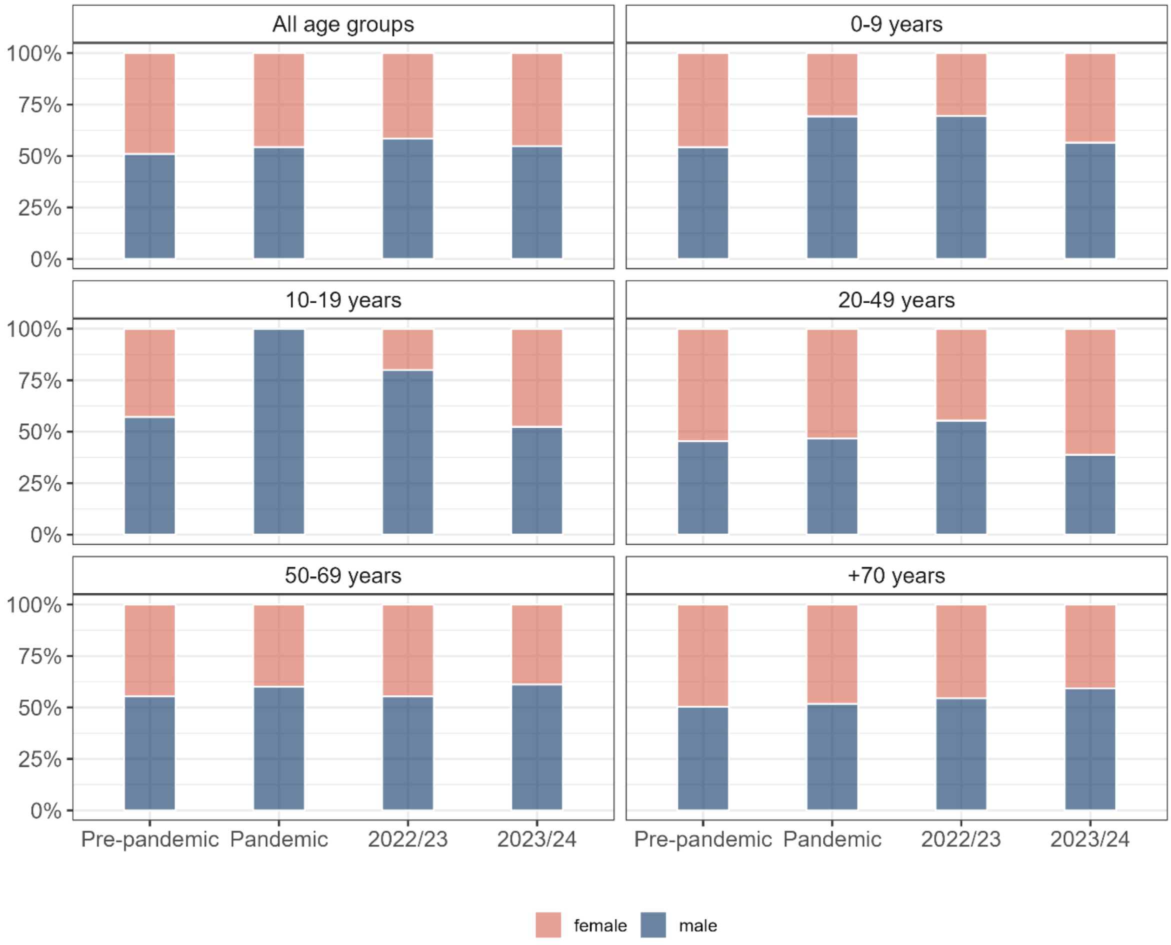

Figure S2: Proportions of male and female cases among all invasive group A streptococcus (iGAS) by age groups and study periods. Pre-pandemic period from 1st March 2015 to 28th February 2020. Pandemic period from 1st March 2020 to 28th February 2024. 2022/23 period from 1st March 2022 to 28th February 2023. 2023/24 period from 1st March 2023 to 29th February 2024.

Table S2. Number of invasive group A streptococcus (iGAS) isolates with emm-type data by age groups and study periods, 1 March 2019 – 29 February 2024.

					Late/Post-pandemic[3]			
**Characteristics**	**Pre-pandemic[1]**		**Pandemic[2]**		**2022/2023**		**2023/2024**	
**Age groups**	N	N_{emm}	N	N_{emm}	N	N_{emm}	N	N_{emm}
0 - 9	105	15	13	10	49	43	85	51
10 - 19	28	6	6	5	5	4	21	3
20 - 49	291	31	75	62	65	61	116	19
50 - 69	316	36	45	39	56	48	139	24
+70	396	41	58	51	68	63	194	37
Total	1 136	129	197	167	24	219	421	134

[1]Pre-pandemic period from 1st March 2019 to 28th February 2020. [2] Pandemic: COVID19 pandemic period from 1st March 2020 to 28th February 2022. [1]Late/Post-pandemic period from 1st March 2022 to 29th February 2024. N; total number of isolates. Nemm; number of emm-typed isolates

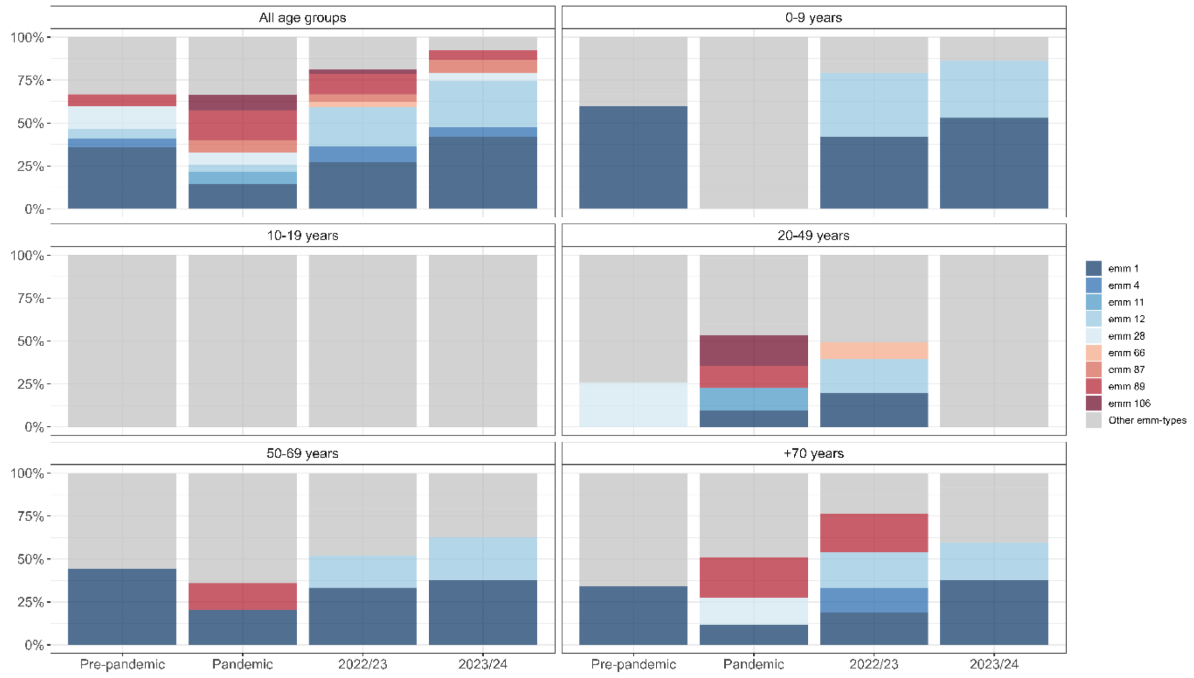

Figure S3: Proportions of emm types among invasive group A streptococcus (iGAS) isolates with molecular data (n = 649) by study periods and age groups, Norway. The graph presents the nine most common emm-types in Norway (N>5 per age group and study period). Other emm-types aggregates all emm-types where N <=5 per age group and study period. Pre-pandemic period from 1st March 2019 to 28th February 2020. COVID-19 pandemic period from 1st March 2020 to 28th February 2022. 2022/23 period from 1st March 2022 to 28th February 2023. 2023/24 period from 1st March 2023 to 29th February 2024.

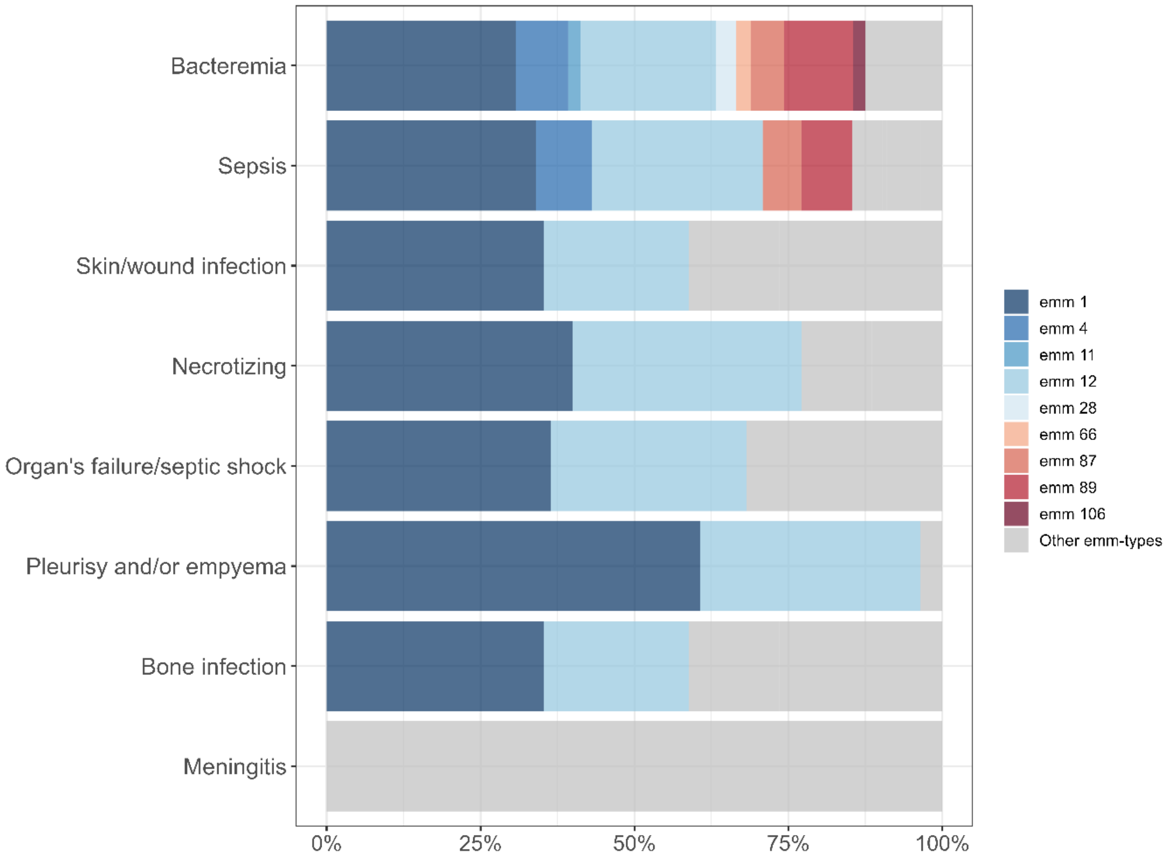

Figure S4. Notified clinical manifestations of invasive group A streptococcus (iGAS) infections by emm-types for cases reported from 1st March 2022 and 1st March 2024 in Norway. The graph presents the nine most common emm-types in Norway (N>5 per clinical manifestation). Other emm-types aggregates all emm-types where N <=5 per clinical manifestation.

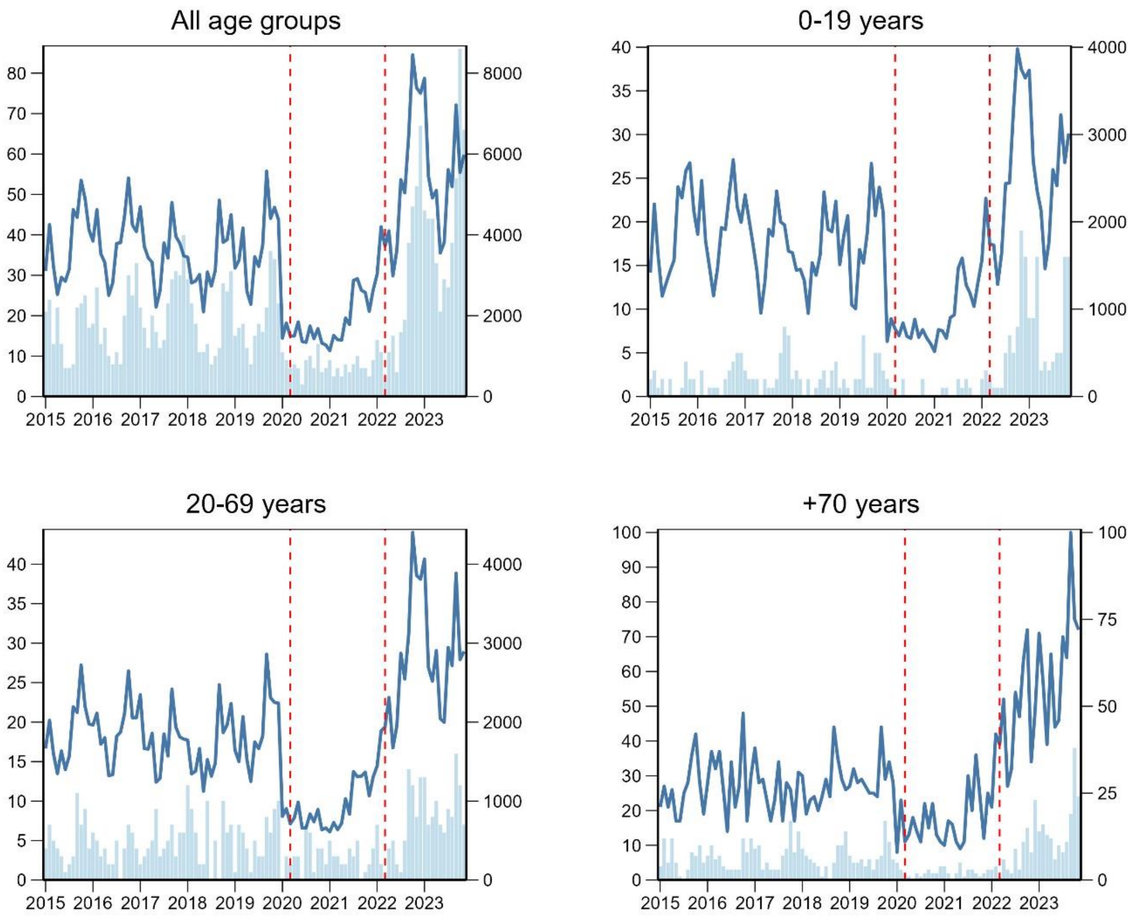

Figure S5. Monthly number iGAS notifications (light blue bars, primary x-axis) and monthly number of primary care consultations for with ICPC-2 code R72 - streptococcal pharyngitis-tonsillitis (dark blue line secondary x-axis) and by age group from 1st March 2022 and 1st March 2024 in Norway.

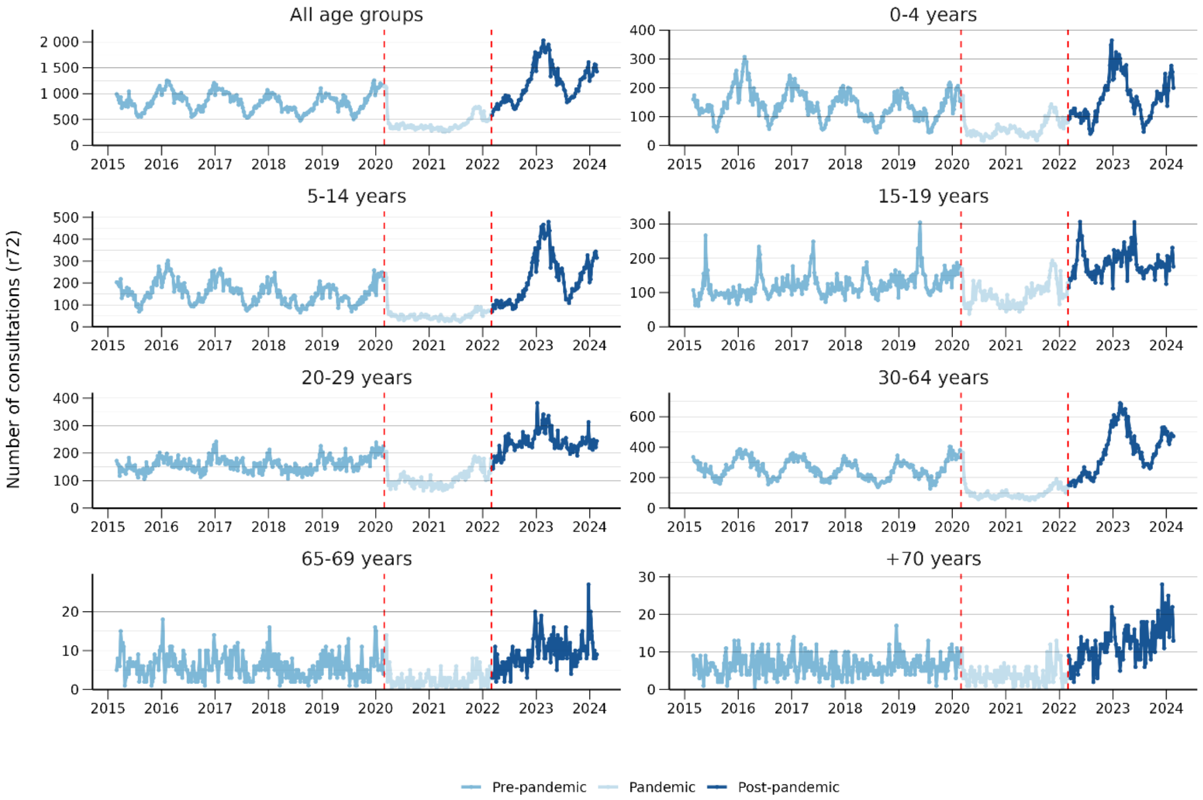

Figure S6. Weekly number of primary care consultations for with ICPC-2 code R72 (streptococcal pharyngitis-tonsillitis) reported from 1st March 2022 and 1st March 2024 in Norway. Pre-pandemic period from 1st March 2015 to 28th February 2020. Pandemic period from 1st March 2020 to 28th February 2022. Late/Post-pandemic period from 1st March 2022 to 29th February 2024. Vertical red dotted lines represent the start and end of the pandemic period.
